# Quantitative Structure–Activity Relationship Study of Cathepsin L Inhibitors as SARS-CoV-2 Therapeutics Using Enhanced SVR with Multiple Kernel Function and PSO

**DOI:** 10.3390/ijms26178423

**Published:** 2025-08-29

**Authors:** Shaokang Li, Zheng Li, Peijian Zhang, Aili Qu

**Affiliations:** 1 College of Computer Science and Technology, Qingdao University, Qingdao 266071, China; 2246282301@qdu.edu.cn (S.L.); 2942449286@qdu.edu.cn (Z.L.); zpj@qdu.edu.cn (P.Z.); 2 School of Economics, Qingdao University, Qingdao 266071, China

**Keywords:** support vector regression, Cathepsin L inhibitor, SARS-CoV-2, particle swarm optimization, molecular docking

## Abstract

Cathepsin L (CatL) is a critical protease involved in cleaving the spike protein of severe acute respiratory syndrome coronavirus 2 (SARS-CoV-2), facilitating viral entry into host cells. Inhibition of CatL is essential for preventing SARS-CoV-2 cell entry, making it a potential therapeutic target for drug development. Six QSAR models were established to predict the inhibitory activity (expressed as IC_50_ values) of candidate compounds against CatL. These models were developed using statistical method heuristic methods (HMs), the evolutionary algorithm gene expression programming (GEP), and the ensemble method random forest (RF), along with the kernel-based machine learning algorithm support vector regression (SVR) configured with various kernels: radial basis function (RBF), linear-RBF hybrid (LMIX2-SVR), and linear-RBF-polynomial hybrid (LMIX3-SVR). The particle swarm optimization algorithm was applied to optimize multi-parameter SVM models, ensuring low complexity and fast convergence. The properties of novel CatL inhibitors were explored through molecular docking analysis. The LMIX3-SVR model exhibited the best performance, with an $R^{2}$ of 0.9676 and 0.9632 for the training set and test set and RMSE values of 0.0834 and 0.0322. Five-fold cross-validation $R_{5-fold}^{2}$ = 0.9043 and leave-one-out cross-validation $R_{loo}^{2}$ = 0.9525 demonstrated the strong prediction ability and robustness of the model, which fully proved the correctness of the five selected descriptors. Based on these results, the IC_50_ values of 578 newly designed compounds were predicted using the HM model, and the top five candidate compounds with the best physicochemical properties were further verified by Property Explorer Applet (PEA). The LMIX3-SVR model significantly advances QSAR modeling for drug discovery, providing a robust tool for designing and screening new drug molecules. This study contributes to the identification of novel CatL inhibitors, which aids in the development of effective therapeutics for SARS-CoV-2.

## 1. Introduction

The coronavirus illness of 2019 (COVID-19) was caused by the positive-sense, single RNA virus known as the severe acute respiratory syndrome coronavirus 2 (SARS-CoV-2) [1], which first appeared in December 2019 and then spread throughout the world [2,3,4,5]. International public health infrastructure and economic productivity have been negatively impacted by the estimated 777 million verified COVID-19 illnesses and 7.07 million deaths worldwide as of early April 2024 [6,7,8,9,10]. Currently, the principal preventative strategy is still vaccination against inactivated viruses [11]. Nevertheless, this method can only stop infection; it cannot stop the virus from infecting people who are already infected. Additionally, some immunocompromised populations may be in danger from vaccinations [12].

Currently available treatments for SARS-CoV-2 include remdesivir [13] and molnupiravir [14], which target RNA-dependent RNA polymerases [13,14]. These medications do, however, have serious drawbacks, including lack of specificity and severe responses. The discovery of safe, oral bioavailable anti-SARS-CoV-2 medications is therefore desperately needed, especially those that can lessen the virus’s possible long-term effects.

In the latest research, an elevated level of circulating cathepsin L (CatL) is correlated [15] with COVID-19 disease progression and severity. Emerging evidence posits that the SARS-CoV-2 Omicron variant may exhibit a predilection for the CatL-mediated endosomal pathway for cellular entry [16,17]. Thus, CatL can be a potential target against SARS-CoV-2. A promising therapeutic approach for COVID-19 could involve the attenuation of CatL activity to obstruct the viral entry into host cells.

The literature [18] indicates that a new class of peptidomimetic analogues (PDAs) was recognized as an effective cathepsin L (CatL) inhibitor, and peptidomimetic aldehyde exhibited notable efficacy in suppressing CatL enzymatic function. To find compounds with improved therapeutic effectiveness and fewer adverse effects, more research should be performed on a number of PDAs that structurally resemble peptidomimetic aldehyde. The potency of inhibition toward CatL can be quantified using the IC_50_ metric, representing the inhibitor concentration required to decrease CatL activity by 50%. As such, determining IC_50_ values for various PDAs is pivotal in the process of identifying more effective and safer CatL-targeting drug candidates.

Due to the fact that the traditional IC_50_ measurement method consumes a lot of manpower and material resources, more efficient and economical methods are needed.

The quantitative structure–activity relationship (QSAR) [19,20] is a technique utilized to define the link between molecular structure and specific biological activities using mathematical modeling. The fundamental tenet of QSARs is that a compound’s physical characteristics and bioactivity are determined by its chemical structure [21]. The main goal is to develop a quantitative relationship model between a compound’s structural features and biological activity in order to forecast the biological activity or other pertinent qualities of novel compounds and to direct its synthesis and design. The physical and chemical characteristics of substances can be described by molecular descriptors in QSAR models [22,23]. Therefore, by employing mathematical techniques built from carefully selected chemical descriptors, a QSAR makes it possible to estimate biological activity in new substance [24,25].

QSAR models contribute to lowering research expenditures and enhancing productivity in drug development, thereby offering a reliable strategy for estimating the IC_50_ of CatL inhibitors and efficiently identifying promising candidate compounds.

Six models based on four modeling methodologies were created and compared in this study. These included the statistical method heuristic method (HM), the evolutionary algorithm gene expression programming (GEP), the ensemble learning method random forest (RF), and the kernel-based machine learning algorithm support vector regression (SVR). For SVR, three kernel structures were explored: a single kernel using the radial basis function (RBF), a dual kernel combining linear and radial basis functions (LMIX2-SVR), and a triple kernel integrating linear, radial basis, and polynomial functions (LMIX3-SVR). These models were constructed to predict the IC_50_ values of CatL inhibitors. In the SVR models, the single kernel employed the radial basis function, the double kernel combined the radial basis and polynomial functions linearly, while the triple kernel incorporated radial basis, polynomial, and linear functions through linear integration. LMIX3-SVR outperformed other SVR models in terms of performance and robustness, highlighting the usefulness of its integrated triple kernel design in assisting creative drug development initiatives. Furthermore, in order to facilitate the logical design and screening of new chemical entities, the created models were applied to docking experiments and molecular activity prediction. Five new CatL inhibitors stood out from 578 new designed CatL inhibitors. According to these experimental results, the developed compounds performed better, which supports the idea that they could be used as better choices in the context of target-specific inhibition. The study offers new compounds that target and inhibit CatL, providing important information for the creation of antiviral medications that combat SARS-CoV-2.

## 2. Results

### 2.1. HM

**Figure 1:**
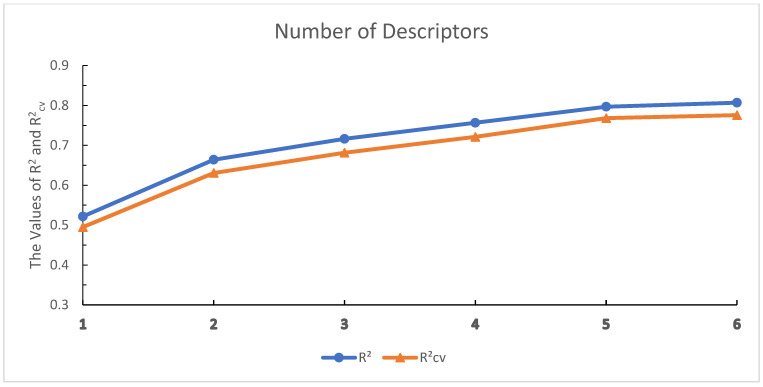
Influences of the number of descriptors on $R^{2}$, $R_{cv}^{2}$.

A total of 604 molecular descriptors were computed using the CODESSA 2.64 software (https://revvitysignals.com accessed on 10 January 2025). To identify molecular descriptors most associated with CatL inhibitory activity, a series of linear models were constructed using progressively more descriptors. Figure 1 depicts the impact of descriptor quantity on both $R^{2}$ and $R_{cv}^{2}$.

As shown in Figure 1, the $R^{2}$ and $R_{cv}^{2}$ improve as the number of descriptors increases. However, when the number of descriptors reached five, increasing the number of descriptors did not significantly improve the $R^{2}$ and $R_{cv}^{2}$. Therefore, the five descriptors in the HM model can be regarded as the most critical ones. Table 1 lists the five selected molecular descriptors along with their physicochemical interpretations, while Table 2 presents their correlation coefficients.

**Figure 2:**
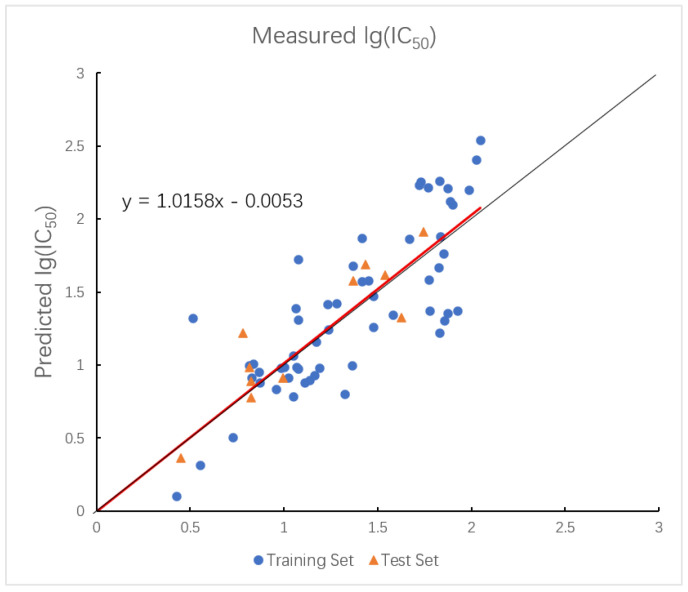
Plot of measured and predicted lg (IC_50_) by HM model. The black line is the y = x line, and the red line is the regression line.

In the HM model, the $R^{2}$ values for the training and test sets are 0.8000 and 0.8159, with corresponding RMSE values of 0.0658 and 0.0764. Moreover, the plot of measured and predicted lg (IC_50_) by the HM model with five descriptors is shown in Figure 2.

In summary, the HM model provides moderate prediction accuracy and good interpretability, but its linear nature limits its ability to capture complex nonlinear relationships.

Equation (1) is the HM model’s mathematical expression:

(1) $$-\mathrm{lg}\left({IC}_{50}\right)=-68.732-37.67\cdot RNR+0.204\cdot HDH2\left(QCP\right)-4.902\cdot \frac{YS}{YR}+25.354\cdot MPPBO+0.242\cdot MEERCOB$$

### 2.2. XGBoost

**Figure 3:**
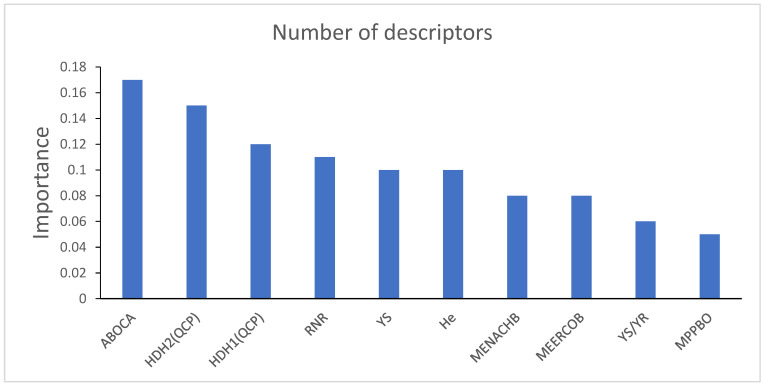
Importance of ten descriptors selected by XGBoost.

Using a nonlinear method to select descriptors can better capture nonlinear relationships between data [26]. Therefore, the correctness of descriptors selected by HM is verified by the nonlinear method XGBoost. All descriptors calculated from CODESSA were exported and preprocessed. The split gain for each descriptor was then calculated to determine the importance of the descriptor. Table 3 shows the physical–chemical meanings of the top ten descriptors selected by XGBoost. Figure 3 shows the ten descriptors with highest importance. Table 4 shows the correlation coefficients between the ten most important descriptors screened by XGBoost.

However, the HM method selects descriptors by considering the correlation between them, while XGBoost only ranks descriptors based on the importance score calculated from the split gain method, completely ignoring the correlation between the descriptors. This leads to a key issue that a considerable proportion of correlation coefficients between the top ten descriptors selected by XGBoost exceed 0.6, as clearly demonstrated in Table 4. When these highly relevant descriptors are removed, the resulting subset is exactly consistent with the descriptors chosen by the HM method.

XGBoost selection results based on split gain importance strongly validate the rationality of the HM descriptor selection method. Thus, while XGBoost serves for initial importance evaluation, HM proves superior for obtaining nonredundant descriptor sets, justifying its use in this study.

### 2.3. GEP

Five selected descriptors were input into APS 2.9 software (http://www.gepsoft.com accessed on 2 April 2025) to construct the GEP model.

**Figure 4:**
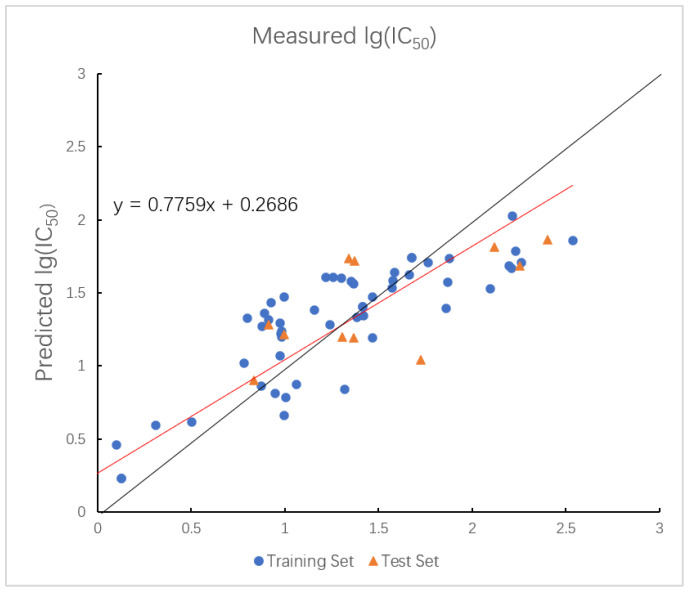
Plot of measured and predicted lg (IC_50_) by GEP model. The black line is the y = x line, and the red line is the regression line.

In the GEP model, the $R^{2}$ values for the training and test sets are 0.7637 and 0.7798, with corresponding RMSE values of 0.3394 and 0.2400. The plot of measured and predicted lg (IC_50_) by the GEP model with five descriptors is shown in Figure 4. The nonlinear model established by GEP is expressed as Equation (2).

While GEP is capable of modeling nonlinear patterns and yields interpretable equations, its prediction accuracy was relatively low, suggesting limited generalization capacity.

(2) $$\begin{array}{ll} \mathrm{lg}\left({IC}_{50}\right) & =fmod(\sin\left(\mathrm{lg}\left(d\left[0\right]\cdot d\left[0\right]\right)\right),d[0])+fmod(floor((fmod\frac{\left(d\left[1\right],d\left[4\right]\right)}{\mathrm{lg}\left(d\left[0\right]\right)})))\\ & \;\;\;\;+fmod(fabs(d\left[4\right],\sin\left(d\left[1\right]\right)))\\ & \;\;\;\;+fabs(\tan(\sin\left(\cos\left(d\left[1\right]\right)\right))\\ & \;\;\;\;+\sin(\tan(\cos\left(\left(\left(pow\left(d\left[0\right],d\left[3\right]\right)\cdot \tan\left(d\left[0\right]\right)\right)\cdot \left(d\left[1\right]\cdot d\left[4\right]\right)\right)))+floor(floor\left(d\left[0\right]\right)\right) \end{array}$$

### 2.4. RF

When using RF regression, the following four important parameters were determined [27].

1. The number of decision trees in the forest (NT): This number is positively correlated with model performance and computational complexity.
2. The minimum number of samples required for node splitting (MS): It serves to constrain the minimum size of leaf nodes, avoiding mitigating overfitting.
3. The maximum depth of a single tree (MD): This parameter limits the complexity of the tree and prevents overfitting.
4. The minimum number of samples required for a leaf node (ML): This parameter will prevent the appearance of leaves with a very small number of samples, improving the generalization ability of the model.

**Figure 5:**
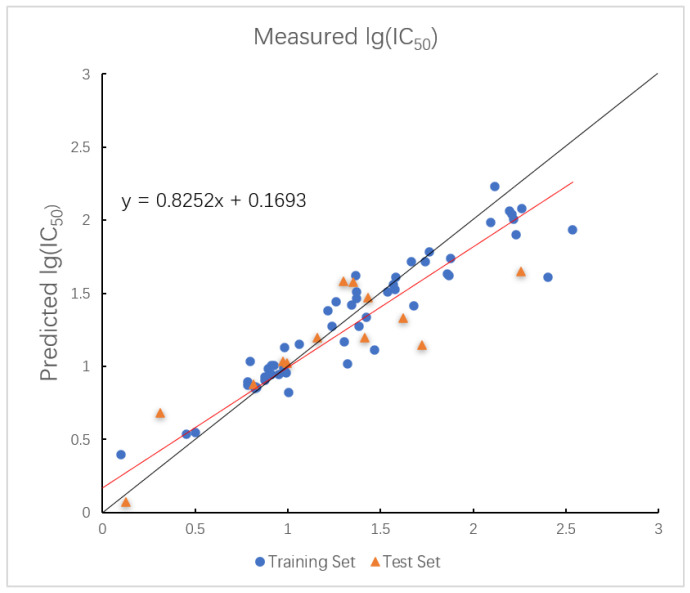
Plot of measured and predicted lg (IC_50_) by RF model. The black line is the y = x line, and the red line is the regression line.

The optimal parameters for the RF model are shown below. In the RF model, the $R^{2}$ values for the training and test sets are 0.9617 and 0.7781, with corresponding RMSE values of 0.1321 and 0.3089. The plot of measured and predicted lg (IC_50_) by the RF model with five descriptors is shown in Figure 5.

The RF model demonstrated high training accuracy but showed signs of overfitting with lower test set performance, indicating weaker robustness.

$$\left(NT,MS,MD,ML\right)=\left(327,8,16,3\right)$$

### 2.5. RBF-SVR

RBF is universal, flexible, and efficient and is often used as a kernel function. And the effect of RBF mainly depends on the penalty factor C and scale factor γ. C controls the amount of regularization applied to the data. Higher values of C lead to better fit on the training data, while simultaneously raising the likelihood of overfitting [28]. γ controls the width of the RBF nucleus. A larger γ value makes the kernel function more sensitive to distance, and a smaller γ value makes the kernel less sensitive to distance. In addition, the effectiveness of the SVR is also affected by ε. The ε parameter determines how sensitive the model is to input noise. The PSO algorithm is used in the process of parameter optimization to accelerate the convergence speed. The best-performing RBF parameter combination obtained via PSO is listed below.

$$\left(C,\varepsilon ,\gamma \right)=\left(4.913,0.014,17.602\right)$$

**Figure 6:**
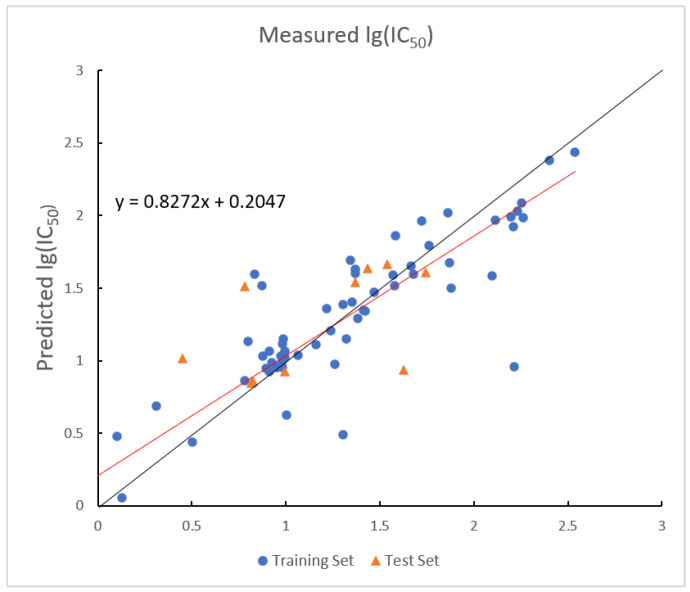
Plot of measured and predicted lg (IC_50_) by RBF-SVR model. The black line is the y = x line, and the red line is the regression line.

The plot of measured and predicted lg (IC_50_) by the RBF-SVR model with five descriptors is shown in Figure 6. The $R^{2}$ of the training set and the test set in the model are 0.9431 and 0.8971, and the RMSE are 0.0063 and 0.0614.

The RBF-SVR demonstrates universal approximation capability, enabling effective modeling of complex nonlinear relationships between molecular descriptors and bioactivity without requiring prior knowledge of data structure. This property receives empirical validation through the model’s training performance metrics, specifically achieving a coefficient of determination value of 0.9431 and maintaining an RMSE of 0.0063. The kernel exhibits high learning efficiency by successfully capturing intricate patterns using only five descriptors while preserving computational feasibility.

The RBF-SVR demonstrates weaker generalization capability, as evidenced by a significant performance gap between training and test sets. While the model achieves strong predictive accuracy on the training data, with an $R^{2}$ of 0.9431, its performance declines to an $R^{2}$ of 0.8971 on the test set. This 4.9% reduction in explanatory power indicates overfitting to training-specific patterns. This divergence implies excessive fitting to training-specific noise patterns, attributable to kernel sensitivity to bandwidth parameter γ.

### 2.6. LMIX2-SVR

However, a 0.046 higher $R^{2}$ value of the training set than that of test set indicates overfitting of the RBF-SVR model, and a balance between the model’s learning capacity and generalization ability needs to be maintained. Therefore, the combination of RBF kernel function with linear kernel function is intended to balance the model’s learning ability against its generalization capacity. According to Equation (9), a new parameter α is added to indicate the proportion of the RBF kernel of the double-kernel function. The optimal parameter set for the dual-kernel function, obtained via PSO, is presented below.

$$\left(C,\varepsilon ,\sigma ,\alpha \right)=\left(16.324,0.997,22.840,0.863\right)$$

**Figure 7:**
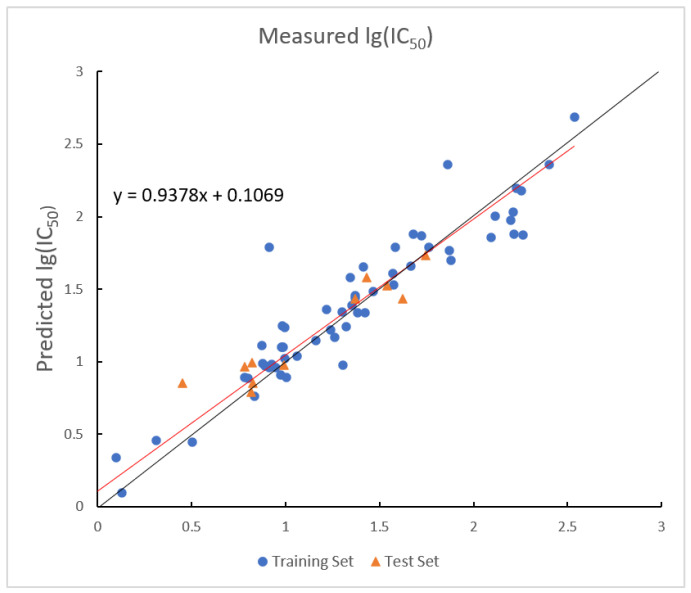
Plot of measured and predicted lg (IC_50_) by LMIX2-SVR model. The black line is the y = x line, and the red line is the regression line.

The optimal prediction results of the model established by the LMIX2-SVR are shown in Figure 7. The $R^{2}$ values of the training set and the test set in the model are 0.9671 and 0.9410, and the RMSE values are 0.0045 and 0.1199.

The LMIX2-SVR model demonstrates robust predictive performance, achieving a training set $R^{2}$ value of 0.9671 and maintaining a test set $R^{2}$ value of 0.9410. These results indicate superior generalization capability when applied to out-of-sample data. The dual-kernel architecture effectively combines the linear kernel stability in handling low-dimensional linear relationships with the RBF kernel strength in modeling high-dimensional nonlinear patterns. Notably, while the linear kernel ensures out-of-sample robustness, the RBF kernel successfully captures complex feature interactions. This combination enables the model to maintain balanced adaptability across heterogeneous data structures, as demonstrated by a test RMSE value of 0.1199 despite its high training accuracy.

The double-kernel architecture in this model requires simultaneous optimization of multiple hyperparameters, which significantly increases computational overhead. This complexity raises overfitting risks, as shown by the 0.0261 generalization gap between the training set $R^{2}$ value of 0.9671 and test set $R^{2}$ value of 0.9410. Parameter selection sensitivity in the RBF kernel may worsen performance instability in noisy or high-dimensional domains, where improper calibration could disproportionately amplify prediction errors. Additionally, the linear–RBF combination needs careful weighting to prevent either kernel from dominating. If the kernel contributions are not properly balanced, model performance may degrade when handling datasets that have sharp transitions between linear and nonlinear patterns.

### 2.7. LMIX3-SVR

The results demonstrate that, in the LMIX2-SVR model, the $R^{2}$ value for the training set remains more than 0.026 higher than that of the test set, suggesting insufficient generalization performance. To address this limitation, the polynomial kernel function was incorporated into the mixed kernel configuration of LMIX3-SVR in order to enhance the model’s generalization ability. Compared to LMIX2-SVR, parameters β and D are additional, which represent the proportion of polynomial kernel function of triple kernel function and the order of the polynomial. The optimal LMIX3-SVR parameter set obtained through PSO is listed below.

$$\left(C,\varepsilon ,\gamma ,\alpha ,\beta ,D\right)=(123.695,1.000,23.221,0.324,0.515,0.161)$$

**Figure 8:**
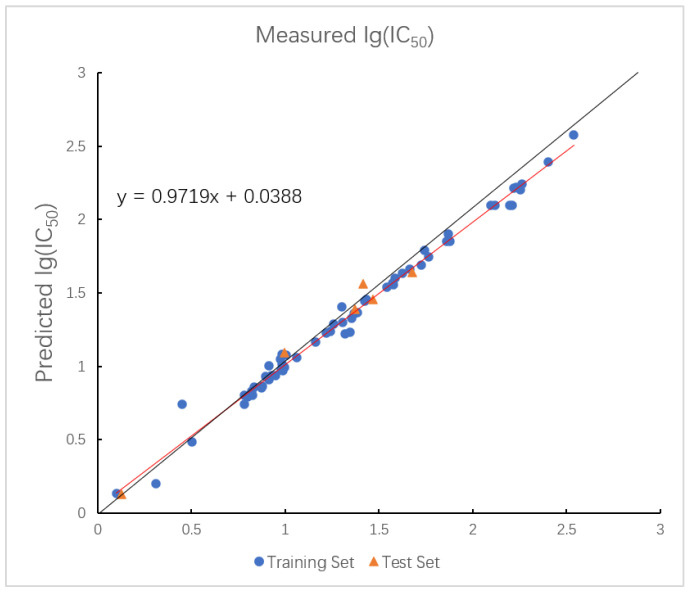
Plot of measured and predicted lg (IC_50_) by LMIX3-SVR model. The black line is the y = x line, and the red line is the regression line.

In the optimized triple-kernel SVR model, the assigned weights for the RBF, polynomial, and linear components were 0.324:0.515:0.161, which indicates that the polynomial kernel function plays an important role in implementing the inner product operation of the kernel function. Figure 8 shows the optimal prediction result of the LMIX3-SVR model. The $R^{2}$ values of the training set and the test set in the model are 0.9676 and 0.9632, and the RMSE values are 0.0834 and 0.0322. Although the fitting curve appears tight, the minimal difference between training and test $R^{2}$ values, together with cross-validation and external validation results, indicates that the model achieves high accuracy without overfitting.

The triple-kernel architecture achieves exceptional generalization performance, demonstrated by a test $R^{2}$ of 0.9632 and test RMSE of 0.0322. The synergistic integration of kernels enhances adaptability to diverse data characteristics: the linear kernel stabilizes low-dimensional projections, the RBF kernel captures complex nonlinear patterns, and the polynomial kernel enables high-dimensional feature representation. The minimal training–test $R^{2}$ gap of 0.0044 confirms balanced learning capacity and robustness across heterogeneous datasets.

Computational complexity escalates significantly due to concurrent optimization of six interdependent hyperparameters, with high-dimensional polynomial operations substantially increasing training time and resource demands.

### 2.8. Design of New CatL Inhibitors

The factors affecting the IC_50_ of the CatL inhibitor were obtained by analyzing the molecular descriptors of the HM model. The nonnormalized coefficients in Table 1 reflect the slope of the regression equation for each independent variable, indicating how much the dependent variable IC_50_ changes in response to variations in each predictor. The five descriptors were ordered according to their importance: RNR > HDH2(QCP) > YS/YR > MPPBO > MEERCOB.

1. “RNR” refers to the relative number of cyclic structures in a compound. Decreasing this value significantly reduces the IC_50_ value [29].
2. “HDH2(QCP)” describes the hydrogen donor charged solvent-accessible surface area. Increasing this value slightly increases the IC_50_ value [30].
3. “YS/YR” is the areas of the shadows S_2_ of the molecule as projected on the YZ plane. Increasing this value slightly lowers the IC_50_ value [31].
4. “MPPBO” relates to the strength of intramolecular bonding interactions and characterizes the stability of the molecules. A positive coefficient indicates that increasing the value raises the IC_50_ value [32].
5. “MEERCOB” refers to the maximum electron–electron repulsion force of a carbon–oxygen bond. The coefficient of “MEERCOB” indicates that increasing its value slightly increases the IC_50_ value [30].

**Figure 9:**
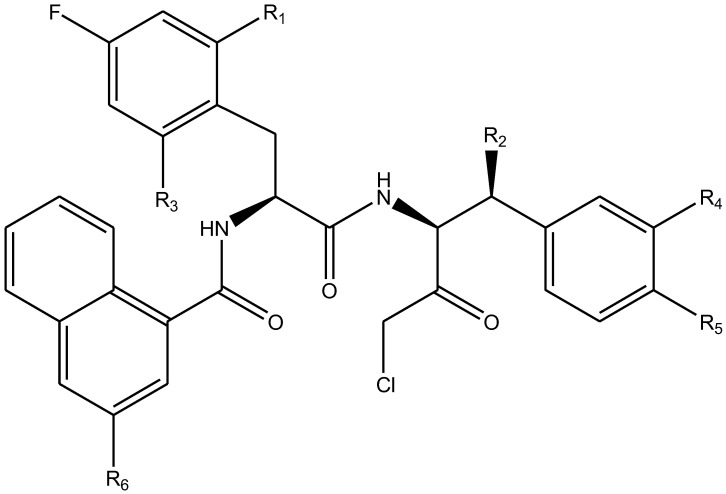
The design strategy mainly focused on the R region of compound **71**.

In summary, the linear model and interpretation of molecular descriptors have revealed several critical factors influencing the compound activity. In order to design new and ideal CatL inhibitors, the rational approaches include reducing the number of cyclic structures in the compounds and improving intermolecular stability. Therefore, increasing the number of hydrogen bonds may favor the enhancement of the effect. Compound 71 is the most potent compound in the literature because it has the lowest IC_50_ value [33], and its structural composition can be modified based on these factors. The main adjustment positions of the molecular structure are shown in Figure 9.

To attenuate the polar interactions between atoms and improve the distribution of various charges, functional groups such as halogens, carboxyls, hydroxyls, hydrocarboxyls, aldehydes, and amino groups were added and randomly combined at the R1 to R6 positions. Based on the descriptor analysis in the HM model, a group of 578 molecules was designed.

Descriptors of the newly designed molecules were computed using CODESSA, and their IC_50_ values were subsequently predicted using the HM model. When the predicted IC_50_ of a compound was below that of compound **71**, it was retained for further evaluation and docking via the Property Explorer Applet (PEA). In the end, five compounds had lower IC_50_ values than compound **71**, and Table 5 shows the predicted IC_50_ values and docking total scores of the newly designed CatL inhibitors.

### 2.9. Property Prediction of New CatL Inhibitors

Property Explorer Applet (PEA) (https://www.organic-chemistry.org accessed on 16 April 2025) was applied to analyze the properties of new compounds. The applet offers real-time prediction of physicochemical properties and assesses potential toxicity risks for user-defined chemical structures. The tool evaluates multiple compound properties, such as partition coefficient, water solubility, topological polar surface area (TPSA), drug likeness, and more.

The partition factor P is described as a particular proportion of solute concentrations between two solvents, and LogP is the logarithm of this ratio. LogP denotes the log-transformed ratio of a compound’s solubility in n-octanol versus water and serves as a standard indicator of hydrophilicity. Compounds with LogP values ≤ 5.0 are more likely to possess good absorption characteristics [34].

Water solubility plays a crucial role in determining the intestinal uptake and cellular distribution of compounds. Enhanced solubility in aqueous environments generally leads to improved absorption of the designed molecules.

TPSA is the total surface area of polar molecules within a compound. The main contribution of TPSA is that it greatly reduces the likelihood of molecules crossing the membrane [35].

Drug likeness is used to evaluate the comparison of objects in terms of bioavailability [36]. Drug score combines the above characteristics into an overall score and is an important criterion for evaluating a compound’s overall potential to become a drug candidate. Table 6 shows the calculated properties of the newly designed compounds by PEA and corresponding predicted IC_50_ by the HM model.

### 2.10. Applicability Domain Analysis

**Figure 10:**
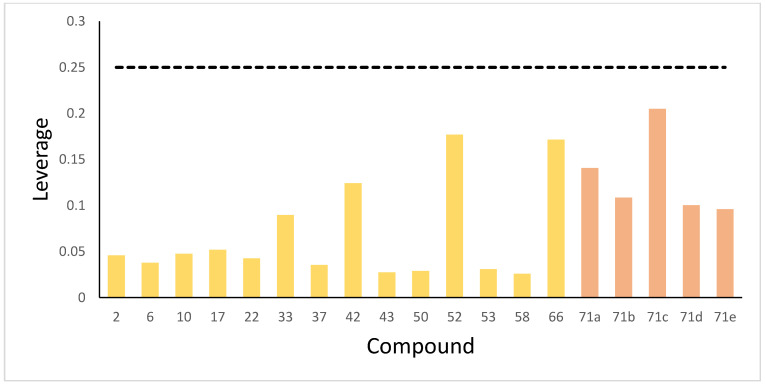
The leverage of test set compounds and newly designed compounds. The dotted line is the warning threshold.

The reliability of predictions was assessed using the applicability domain (AD), defined herein via the leverage method. Predictions for compounds residing within the AD represent interpolations and are thus reliable. In contrast, predictions for compounds located outside the AD constitute extrapolations, entailing higher uncertainty and consequently reduced reliability. Figure 10 demonstrates that all five newly designed compounds and the entire test set exhibited leverage values under the warning threshold ($h^{*}$ = 0.25), thereby validating the reliability of their predictions. Nevertheless, the current AD scope is constrained by dataset size—a characteristic limitation of supervised machine learning frequently leading to a narrow AD [37]. The future development of novel anti-SARS-CoV-2 agents holds promise for broadening the model’s chemical space coverage, which will expand its applicability domain and ultimately enhance prediction reliability for emerging viral variants.

### 2.11. Molecular Docking of New CatL Inhibitors

To explore the binding affinities of the newly designed CatL inhibitors, molecular docking studies were carried out using Sybyl-X2.1. The receptor, cathepsin L (PDB ID: 7W33), was prepared by removing all water molecules and heteroatoms. The structure was optimized by adding missing hydrogen atoms and assigning Gasteiger charges to ensure a stable conformation suitable for docking. The protein was energy-minimized using the Tripos force field, a method that ensures proper alignment for molecular docking.

For the ligands (inhibitors), 3D structures were constructed, and charges were assigned using the Gasteiger method. The molecules were minimized using the MMFF94 force field to ensure the stability of their conformations.

Docking was performed using the FlexX docking algorithm within Sybyl-X2.1, which is based on a flexible docking approach. This algorithm first aligns the ligand to the receptor’s active site and then allows the ligand to adjust its conformation to fit the binding pocket of the receptor. The algorithm scores the generated docking poses by calculating their interaction energies, considering factors such as hydrogen bonding, hydrophobic interactions, and electrostatic forces between the ligand and the receptor.

**Figure 11:**
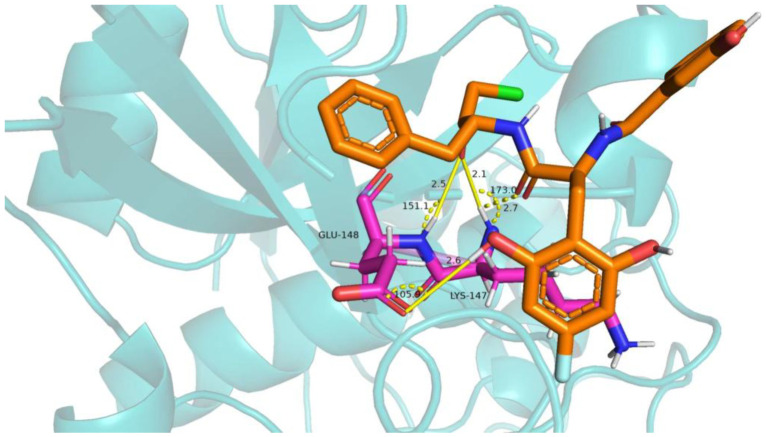
Docking analysis of compound 71d with PDA-related target (PDB ID:7w33).

**Figure 12:**
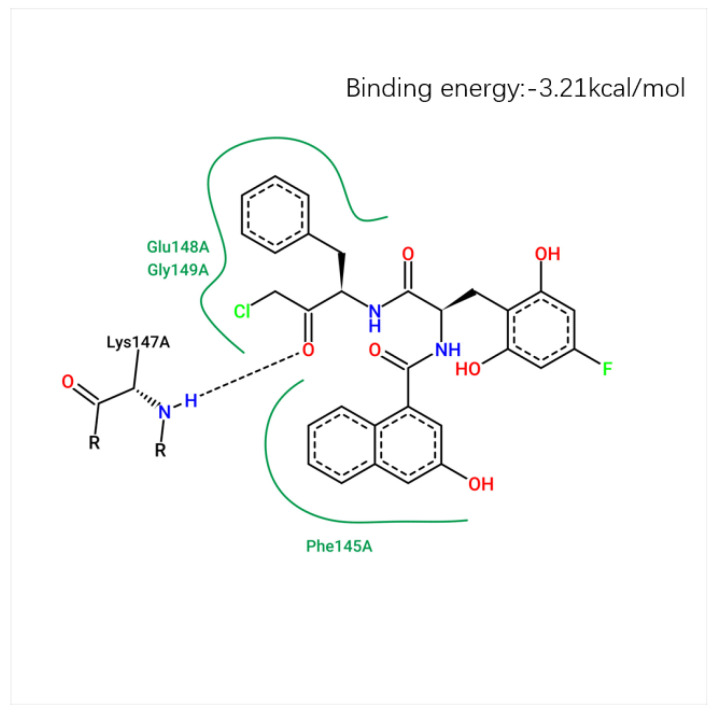
The 2D molecular docking conformation and binding energy.

The docking score, reflecting the binding affinity of the ligand to the target protein, was calculated for each compound. The lower the energy score, the more stable the ligand–receptor interaction. The best docking pose was chosen based on the lowest docking energy, suggesting the most stable binding conformation. The binding conformation of compound **71d** is shown in Figure 11, where the hydrogen bond interactions are visualized with key residues. Figure 12 shows the 2D molecular docking conformation and binding energy.

Among the newly designed compounds, compound **71d** exhibited the most favorable binding affinity, as reflected by a docking score of 5.7972, which was markedly higher than that of compound **71**. According to the predicted docking conformation of compound **71d**, several key hydrogen bonding interactions are established between the ligand and the active site residues of the target protein. Specifically, oxygen atoms within the core structure of **71d** form hydrogen bonds with GLU-148 and LYS-147. The binding site of compound **71d** is located in the active site, specifically in the catalytic region of the protein. The hydrogen bond between compound **71d** and GLU-148 has a bond length of 2.5 Å, with a bond angle of approximately 151.1°. The hydrogen bond with LYS-147 measures 2.7 Å, with a bond angle of about 173.0°. These interactions are crucial for the stability and inhibitory potential of compound **71d**. These interactions are consistent with the binding pattern previously observed in compound **71**, suggesting that compound **71d** retains essential interaction features of its predecessor. In addition, the oxygen atoms introduced through the newly incorporated structural fragment engage in further hydrogen bonding with GLU-148, thereby enhancing the overall interaction strength. The presence of both conserved and newly formed hydrogen bonds contributes to a more stable and favorable binding conformation. This strong molecular recognition between compound **71d** and PDAs supports its potential as a promising lead inhibitor for this protease [27].

## 3. Discussion

As shown in Table 7, the averages of $R_{r}^{2}$ and $R_{5-fold}^{2}$ for all models are much smaller than 0.2 [38], indicating that there are no chance correlations in the models. All optimal predicted results of models based on HM, GEP, RF, RBF-SVR, LMIX2-SVR, and LMIX3-SVR and their $R_{cv}^{2}$ are given in Table 8.

As demonstrated in Table 8, nonlinear models are more adaptable than linear HM models for describing intricate data patterns, but this increased adaptability inevitably makes them more prone to overfitting. As evidenced by the tendency of models such as RF to overfit training data when improperly regularized, this problem can seriously impair generalization performance. Compared to GEP which is often susceptible to converging to a local optimum, SVR offers a distinct advantage for its convex quadratic-based optimization, ensuring that the local optimal solution coincides with the global optimum. In addition, SVR, as opposed to RF, enables the selection and adjustment of kernel functions to better suit the properties of the data and adjust to different distribution patterns. In contrast to RBF-SVR, the difference in $R^{2}$ between the training set and test set for the LMIX2-SVR model decreases significantly, from 0.04600 to 0.00221. This substantial reduction highlights the critical role of the linear kernel function in improving the generalization ability of the LMIX2-SVR model. Compared to LMIX2-SVR, the values for both the training set and test set in the LMIX3-SVR model show a modest increase of 0.0005 and 0.00222, respectively. Especially, the $R^{2}$ gap between the training set and test set of the LMIX3-SVR model is reduced to a mere 0.0044, the smallest $R^{2}$ difference observed between the training set and test set in all models tested, thereby demonstrating that the incorporation of a polynomial kernel function significantly elevates generalization performance of the LMIX3-SVR model.

Because of the synergy between its kernels, LMIX3-SVR outperformed the other two SVR models using distinct kernel functions in both learning and generalization. Furthermore, the cross-validation results also show that the values predicted by the LMIX3-SVR model are the most suitable for the actual data. As shown in Table 9, the LMIX3-SVR model demonstrates excellent generalization ability, as evidenced by the externally validated parameters $CCC,\;Q_{F1,}^{2}Q_{F2}^{2},$ and $Q_{F3}^{2}$, with values of 0.9839, 0.9651, 0.9793, and 0.9632, respectively. These results indicate that the model maintains consistent accuracy across different datasets.

The remarkable performance of the models suggests the descriptors selected by HM are indeed the most relevant and effective in capturing the underlying patterns in the data, further confirming the validity of the HM descriptor selection process.

Additionally, the docking results provide further validation for the compounds identified by the regression models. The top compounds, which showed the best predicted IC_50_ values from the regression models, demonstrated favorable binding affinities in the molecular docking simulations. These compounds not only showed strong interactions with the target protein but also exhibited consistent docking scores with their predicted biological activity. Moreover, the pharmacokinetic properties and toxicity predictions, such as LogP, solubility, and drug likeness, were aligned with the docking results, indicating that these compounds have promising drug-like characteristics. The integration of the regression model, docking analysis, and property predictions presents a comprehensive approach for identifying effective CatL inhibitors, supporting the potential for these compounds to move forward in drug development.

## 4. Materials and Methods

### 4.1. Dataset

The dataset of 74 CatL inhibitors was collected from the literature [18] between 2005 and 2023 and they are listed in Table 10, Table 11, Table 12 and Table 13. Information on the chemical structures and IC_50_ values of the 74 CatL inhibitors is summarized in the tables, with measurements obtained through consistent methodologies and uniform laboratory settings. The dataset was partitioned into training and testing subsets at a 4:1 ratio using a uniform random sampling technique. The internal training set consisted of 60 compounds, which were used for model training and internal cross-validation. The external test set included 14 compounds, which were used to evaluate the model’s prediction accuracy during external validation. In order to test the robustness of the model, 5-fold cross-validation (5-fold) [39] and leave-one-out cross-validation (LOO) were used.

**Figure 13:**
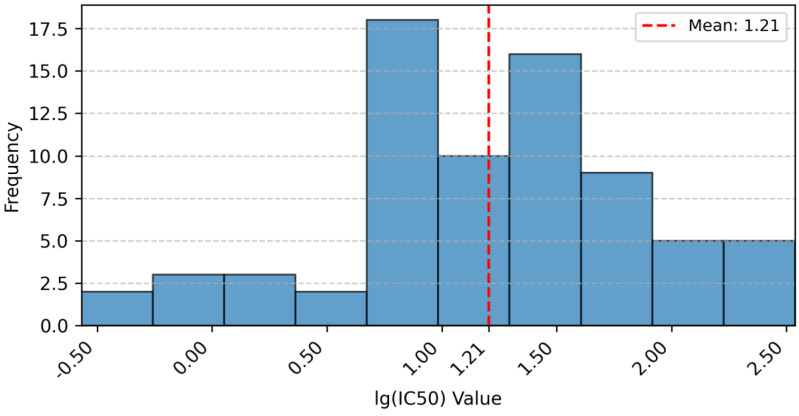
Distribution of lg (IC_50_) Values.

To characterize the dataset’s activity profile, the distribution of measured lg (IC_50_) values was analyzed and visualized (Figure 13). A logarithmic transformation of IC_50_ values was applied for normalization. The histogram shows lg (IC_50_) values range from approximately −0.5 (leftmost bin) to 2.5 (rightmost bin), spanning about three orders of magnitude. This broad range reflects diverse inhibitory potency, critical for QSAR modeling as it enables capturing structure–activity relationships across varying strengths. Values are spread across bins without excessive clustering, confirming sufficient activity diversity. The training set (60 compounds) and test set (14 compounds) exhibit overlapping distributions, ensuring test set representativeness for reliable validation.

### 4.2. Computation of Molecular Descriptors

Molecular descriptors are calculated using CODESSA2.64 (https://revvitysignals.com accessed on 10 January 2025), which provides a wide range of 2D and 3D descriptors. The descriptors used in this study are 2D descriptors and include topology, composition, and electrostatic properties. These descriptors are used to develop QSAR models to predict IC_50_ values of CatL inhibitors.

The steps to calculate the molecular descriptors of a compound are shown below.

The molecular structures of all compounds were first plotted in ChemDraw 8.0 software (https://revvitysignals.com accessed on 1 May 2025) and subsequently aromatized where applicable. Subsequently, the structure of the compound was initially optimized by the MM+ molecular mechanical force field in HyperChem 4.0 software (http://hypercubeusa.com accessed on 17 May 2025) and more accurately by semi-empirical PM3 or AM1 methods [40]. The three-step procedure yielded the conformation with the lowest potential energy and optimal structural stability, thereby contributing to more accurate molecular descriptor calculations. Finally, the files obtained from HyperChem were put into the MOPAC 6.9 [41] software (Stewart Computational Chemistry MOPAC Home Page accessed on 11 May 2025) to produce MNO files, and then the MNO files were utilized as input to the CODESSA 2.64 software to compute five classes of molecular descriptors: constitutional, topological, geometrical, electrostatic, quantum chemical from 604 descriptors [42].

### 4.3. Statistical Parameters

The coefficient of determination, represented by $R^{2}$, was utilized as a measurement of the model’s goodness-of-fit [43]. And the root mean square error (RMSE) was adopted to measure the forecasting accuracy of different models for a particular dataset.

Validating the QSAR model is essential to guarantee its reliability in predicting the biological activity of unknown samples. Model validation includes internal validation, which tests the reproducibility of the model, and external validation, which evaluates the model’s ability to generalize to an independent dataset and its potential for application to novel or external conditions. The internal prediction ability of the model was verified by 5-fold and LOO cross-validation. In addition, four external validation parameters, concordance correlation coefficient (CCC), $Q_{F1}^{2}$, $Q_{F2}^{2}$, and $Q_{F3}^{2}$, were also adopted. Specifically, the concordance correlation coefficient (CCC) assesses reproducibility, a fundamental principle that supports the integrity of the scientific method [44]. The $Q_{F1}^{2}$, $Q_{F2}^{2}$, and $Q_{F3}^{2}$ assess the external prediction performance of the models [44,45,46,47]. Higher values of these metrics indicate better external prediction capability of the model.

y-Randomization was used to test for chance correlations in the process of constructing models. To ensure the randomness of the models, y-randomization was performed for 100 rounds. $R_{r}^{2}$ and $R_{5-fold}^{2}$ of each round were recorded to calculate the averages. These two parameters were used to indicate whether there were any chance correlations in the models.

### 4.4. Linear Model by HM

HM, which has no dataset size limitation, is an effective method for descriptor selection and linear model construction [48]. After obtaining descriptors from CODESSA, preselection is performed to exclude nonuniversal descriptors, constants, highly correlated ones (correlation > 0.8), and those with low F-test or t-values [27,47]. The remaining descriptors are used to build HM linear regression models. The performance of these models depends on the number of selected descriptors, with the optimal number determined by the point at which adding more descriptors does not significantly improve model accuracy. The selected descriptors are then used in subsequent nonlinear models for further analysis.

### 4.5. Calculate Feature Importance by XGBoost

An efficient method is required to optimize nonlinear models and reveal nonlinear interactions among descriptors in complicated datasets. XGBoost is useful for evaluating feature importance by assessing the contribution of each descriptor to model performance.

The coverage method and the split gain method are the two approaches commonly employed in XGBoost to determine feature importance. The split gain approach calculates the increase in prediction accuracy that occurs when decision tree nodes are split using a feature. Higher split gain features are thought to be more significant because they capture more nuanced, nonlinear relationships between descriptors.

The split gain method was applied in this study due to its suitability for uncovering complex relationships within the data. In XGBoost, the importance of molecular descriptors is assessed based on their split gain during the construction of decision trees. Descriptors with higher split gain values, such as hydrophobicity and hydrogen bond counts, show strong associations with inhibitory activity and contribute significantly to predictive performance. The algorithm automatically identifies these key features, while regularization parameters such as lambda and gamma help control model complexity and reduce the risk of overfitting. This approach enhances predictive accuracy and offers valuable insights into the structure–activity relationships of CatL inhibitors.

### 4.6. Nonlinear Model by GEP

Given the inherent complexity and nonlinearity of factors influencing the inhibitory effects of CatL targeting compounds, gene expression programming (GEP) was employed to construct a nonlinear model for predicting their IC_50_ values.

GEP is a new type of adaptive evolution algorithm based on the invention of biological gene structure and function. GEP was developed from genetic algorithms (GAs) and genetic programming (GP) [49,50,51,52], which absorbs the advantages of both but overcomes the shortcomings of both, and its distinctive feature is that it can solve complex problems with simple coding.

GEP uses a unique chromosome-based encoding method that optimizes both the model structure and its parameters simultaneously. This characteristic enables GEP to effectively capture nonlinear relationships between molecular descriptors and biological activity while maintaining strong predictive performance. As a result, GEP is particularly suitable for modeling the complex relationships observed in the CatL inhibitor dataset.

### 4.7. Nonlinear Model by RF

Random forest (RF) regression is an ensemble learning algorithm that constructs multiple decision trees and averages their predictions to perform regression tasks [53]. Each tree is trained on a random subset of samples, which helps reduce overfitting. The final prediction is obtained by averaging the results from all decision trees. RF regression is particularly effective for handling high-dimensional data, missing values, and diverse data distributions. It also provides assessable feature importance and reliable predictions with uncertainty estimation [54]. These advantages make RF regression useful for predicting the IC_50_ value of CatL inhibitors.

The standardized descriptor set was employed to train an optimized RF model, configured with an ensemble of decision trees and constrained leaf node sizes to maintain an optimal balance between predictive accuracy and computational efficiency. Through the training process, the model quantitatively assessed descriptor contributions while capturing complex nonlinear relationships between molecular features and inhibitory potency. Rigorous five-fold cross-validation confirmed model reliability. This dual function model enables accurate IC_50_ prediction for novel CatL inhibitors.

### 4.8. Nonlinear Models by SVR

Support vector regression (SVR), an extension of support vector machine (SVM), seeks a hyperplane that optimally fits continuous data points [55]. SVM maps data to a high-dimensional space to find the best separating hyperplane for classification or regression [56]. Maximizing the margin between samples and the hyperplane enhances prediction accuracy.

SVR differs from SVM in that it focuses on minimizing the overall deviation of all data points from the regression hyperplane, rather than maximizing the margin between the hyperplane and the nearest data points. SVR imported the ε-insensitive loss function to penalize errors beyond a specified threshold ε, which improves robustness and generalization ability of the model. In addition, penalization parameter C serves as a regularization term that regulates the penalty applied to classification errors and slack variables $\xi_{i}$ and $\xi_{i}^{*}$ which are introduced as tolerance allowing some samples at the boundary of classification errors or intervals. The final optimization problem is as follows in Equation (3).

(3) minω,ξi,ξi*12·∥w∥2+CL∑i=1lξi+ξi*s⋅tω⋅xi+b−yi≤ε+ξiyi−ω⋅xi+b≤ε+ξi*ξi,ξi*≥0,i=1,2,…,l.

To simplify the solution for SVR, Lagrange multipliers $\alpha_{j},\;\alpha_{j}^{*}$ are introduced. If the Karush–Kuhn–Tucker conditions are satisfied, the dual problem can be solved by quadrature phase procedures [56]. The final solution is as in Equation (4).

(4) $$f\left(x\right)=\sum_{j=1}^{n}\left(\alpha_{j}-\alpha_{j}^{*})\cdot K(x_{i},x_{j}\right)+b$$

The $K\left(x_{i,}x_{j}\right)$ is the kernel function to be introduced in the next section.

#### 4.8.1. SVR Kernel Function

Standard SVR struggles with nonlinear data due to the reliance on linear decision boundaries. To address this, complex data are mapped to a higher-dimensional space at the cost of increasing complexity and overfitting risk. Kernel functions solve this by efficiently computing inner products in the transformed space, avoiding explicit high-dimensional mappings and simplifying the handling of complex data. Basic kernel functions include the radial basis function (RBF) [57], the linear kernel function, the polynomial kernel function, and the sigmoid kernel function. The initial three kernel functions have been extensively used when establishing models by SVR [58].

However, considering the disadvantages of single kernel functions and the complementarity between popular kernel functions in handling complex datasets, it is necessary and reasonable to construct mixed kernel functions to meet the requirements. In fact, any function kernel K that satisfies the theorem of Mercer can be used as a kernel function, which provides many ways to construct new kernel functions. If $K_{1},$ $K_{2}$ are kernel functions, then:

1 $K=K_{1}+K_{2}$ is a kernel function.
2 $K=\alpha K_{1}+\beta K_{2},\alpha ,\beta \geq 0$ is a kernel function.

More importantly, integrating several basic kernel functions into mixed kernel functions means they are able to complement each other, thus overcoming their respective shortcomings.

The theorem of Mercer is defined as such that if K(x,y) satisfies Mercer’s premise shown in Equation (5) which means that K(x,y) is a semidefinite function [59].

(5) $$\iint K\left(x,y\right)\cdot f\left(x\right)\cdot f\left(y\right)dxdy\geq 0$$

Then it can be expressed in the form of a series expansion of the above-mentioned eigenvalues and eigenfunctions. This implies that K(x,y) can be represented as an inner product in some high-dimensional feature space.

#### 4.8.2. Nonlinear Model

RBF is a universal kernel function that can be employed without prior knowledge of the data [60,61,62,63]. RBF stands out for its good learning ability and efficiency among all basic kernel functions [64]. However, the model exhibits limited generalization performance. The RBF kernel can be expressed as Equation (6).

(6) $$K_{RBF}\left(x,x_{i}\right)=exp\left\{-\frac{{\left\|x-x_{i}\right\|}^{2}}{2\sigma^{2}}\right\}$$

SVR that adopted $K_{RBF}$ as its kernel function is called RBF-SVR.

The linear kernel is the most basic among all kernel functions, as it simply calculates the inner product between two feature vectors. Moreover, the linear kernel function has the advantages of strong learning ability which is restricted to linear relationships and effectively improves the generalization ability when the dataset is linearly separable and low-dimensional. However, since the linear kernel is limited to computing a simple inner product between feature vectors, its modeling capacity is inherently constrained. Consequently, the linear kernel typically achieves lower accuracy compared to more flexible kernel functions when handling nonlinear problems [65], but it efficiently identifies key feature vectors with minimal computational cost. The linear kernel is defined in Equation (7).

(7) $$K_{L}\left(x,x_{i}\right)=\left(x^{T}x_{i}\right)$$

The polynomial kernel function is one of the most widely used nonlinear kernel functions, as it extends the linear kernel by introducing polynomial combinations of feature interactions. Unlike the linear kernel, which only computes a simple dot product, the polynomial kernel maps data into a higher-dimensional space, enabling the learning of complex nonlinear relationships. Moreover, the polynomial kernel function possesses the advantage of adjustable flexibility, governed by its degree parameter, which enables it to model a broad spectrum of decision boundaries spanning approximately linear to highly curved surfaces. This makes it particularly effective when data exhibits polynomial patterns or multiplicative feature dependencies. The polynomial kernel function demonstrates strong generalization performance due to its global approximation properties. However, this comes at the expense of weaker local learning capabilities, as the polynomial kernel tends to smooth over fine-grained variations in the feature space. The polynomial kernel can be expressed as Equation (8).

(8) $$K_{Poly}\left(x,x_{i}\right)={\left[\left(x^{T}x_{i}\right)\right]}^{q}$$

To overcome the limitations of individual kernel functions while preserving their respective advantages, some kinds of mixed kernel functions incorporating different numbers of individual kernel functions were proposed.

The linear kernel function demonstrates strong learning capability and excellent generalization performance for linearly separable and low-dimensional datasets. However, its effectiveness is fundamentally limited to linear relationships. In comparison, the RBF kernel function shows superior learning ability in handling nonlinear and high-dimensional datasets, with relatively weaker generalization performance. To leverage their complementary strengths, a mixed kernel function combining both the RBF and linear kernels was proposed. The mixed kernel function that linearly combines the RBF kernel function and linear kernel function allows the linear kernel to enhance the overall generalization ability while the RBF kernel improves the learning capability for complex patterns, thereby achieving balanced performance across different data characteristics. The form of this new double-kernel function can be expressed as Equation (9).

(9) $$K_{LMIX2}={\alpha \cdot K}_{RBF}+\left(1-\alpha \right){\cdot K}_{L}$$

SVR that adopted $K_{LMIX2}$ as its kernel function is called LMIX2-SVR.

The variable α takes values within the interval [0, 1].

To overcome the limitations of the linear kernel function, which exhibits strong generalization performance only for low-dimensional and linearly separable datasets but struggles with high-dimensional and nonlinear datasets, a mixed kernel function is constructed by incorporating the RBF kernel function, linear kernel function, and polynomial kernel function.

The polynomial kernel further enhances nonlinear modeling by incorporating feature interactions through its adjustable degree parameter. Importantly, the polynomial kernel improves generalization in high-dimensional and nonlinear datasets, effectively compensating for the deficiency of the linear kernel in these scenarios.

The proposed mixed kernel function combines three complementary kernel functions including the linear kernel for low-dimensional linear separability, the RBF kernel for nonlinear pattern recognition, and the polynomial kernel for high-dimensional feature representation. This comprehensive integration achieves optimal balance between learning capacity and generalization ability across diverse data characteristics.

The form of the new triple-kernel function can be expressed as Equation (10).

(10) $$K_{LMIX3}={\alpha \cdot K}_{RBF}+\beta {\cdot K}_{poly}+{\left(1-\alpha -\beta \right)\cdot K}_{L}$$

SVR that adopted $K_{LMIX3}$ as its kernel function is called LMIX3-SVR.

The coefficients α and β are positive and satisfy the constraint α + β ≤ 1.

#### 4.8.3. SVR Model Optimized by Particle Swarm Optimization (PSO)

The performance and generalization capacity of SVR models are highly sensitive to parameter settings. When constructing a model using a triple kernel SVR, six parameters require optimization: the penalty factor C, the insensitive parameter ε, the kernel radius of RBF kernel function σ, the order of polynomial kernel function q, the coefficient of RBF kernel function α, and the coefficient of polynomial kernel function β. Their search scope is as follows. $C\in \left[0.001,100\right]$, $\varepsilon \in \left[0,1.0\right]$, $\sigma \in \left[0.001,50\right]$, $q\in \left\{1,2,3\right\}$, $\alpha \in \left[0,1\right]$, $\beta \in \left[0,1-\alpha \right]$.

The optimization process becomes more difficult as the number of parameters of SVR increases. To address the limitations of conventional parameter tuning techniques such as grid and random search, particle swarm optimization (PSO) was employed to optimize model parameters during the construction of the three SVR-based models.

Particle swarm optimization (PSO), introduced in 1995, draws inspiration from the social behavior of bird flocks searching for optimal routes through shared information. The algorithm initializes particle positions and velocities using random values in a high-dimensional space and iteratively refines them through both individual learning and collective swarm interactions. In PSO algorithms, particles only pass optimal information during iterations. Therefore, PSO has the advantages of fast convergence speed, few parameters, and simple and easy implementation of algorithms.

PSO uses a velocity–position model, where the position and velocity of particles i in D-dimensional solution space can be expressed as Equations (11) and (12).

(11) $$X_{i}=\left(x_{i1},x_{i2},x_{i3},\cdots ,x_{iD}\right)$$

(12) $$V_{i}=\left(x_{i1},x_{i2},x_{i3},\cdots ,x_{iD}\right)$$

The optimal position of particle i is denoted as $P_{ibest}$, the global best position is denoted as $g_{best}$. In each iteration, particles track the $P_{ibest}$, $g_{best}$, and their previous states to adjust the position and velocity at the current moment. The iterative Equations (13) and (14) are as follows.

(13) $$V_{i}(k+1)=w\cdot v_{i}(k)+c_{1}\cdot rand()\cdot (P_{ibest}-x_{i}(k))+c_{2}\cdot rand()\cdot \left(g_{best}-x_{i}\left(k\right)\right)$$

(14) $$X_{i}\left(k+1\right)=X_{i}\left(k\right)+V_{i}\left(k+1\right)$$

$V_{i}(k)$, $X_{i}(k)$, $V_{i}(k+1)$, $X_{i}(k+1)$ are the velocity and position of the particle i at the current time and the next time. rand() is a random number in range $\left[0,1\right]$. $c_{1}$ and $c_{2}$ are learning factors which are usually set to 2. ω is a weight factor, and its value automatically decreases with the iteration of the algorithm to speed up the convergence speed [66]. It is described as Equation (15).

(15) $$\omega =\omega_{min}+\left({iter}_{max}-iter\right)\cdot \frac{\omega_{max}-\omega_{min}}{{iter}_{max}}$$

iter represents the current iteration count, ${iter}_{max}$ denotes the predefined upper limit. The parameters $\omega_{max}$, $\omega_{min}$ correspond to the upper and lower bounds of the inertia weight, respectively [67].

### 4.9. Applicability Domain

The applicability domain (AD) is a vital mechanism for guaranteeing the reliability of predictive models. In this research, the AD served to quantify the uncertainty in predicting the activity of a specific compound. This assessment was accomplished by determining the compound’s structural similarity to those comprising the training set [68]. Specifically, the leverage method defined in Equation (16) was applied.

(16) $$h_{i}=x_{i}^{T}{\left(X^{T}X\right)}^{-1}x_{i}$$

In this context, $x_{i}$ and X denote the descriptor matrix for the test compound i and the training set compounds, respectively, with the superscript  T indicating the matrix transpose. The warning threshold $h^{*}$ is established as 3∗p/n, where p signifies the number of model descriptors and n corresponds to the number of training compounds. Compounds exhibiting a leverage value exceeding this threshold are deemed to lie beyond the AD [69].

### 4.10. Property Prediction and Molecular Docking

Based on a number of carefully chosen molecular descriptors that correspond to the activity of a compound and the ensuing data analysis, the QSAR model is crucial to the creation of new pharmaceuticals. In drug discovery, achieving potent binding affinity to the target is critical for identifying viable drug candidates. Furthermore, key drug-like properties, such as pharmacokinetics and toxicity profiles, are vital factors in determining whether a compound progresses to clinical development [70].

Molecular docking is a key tool in structural molecular biology and computer-aided drug design [71]. The objective of ligand–protein docking is to predict the primary binding mode between a ligand and a known three-dimensional protein. In this study, Sybyl-X 2.1 software was used to explore the potential interactions between the newly designed CatL inhibitors and the target protein at the binding site. The target protein, cathepsin L (PDB code: 7w33), was retrieved from the Protein Data Bank. The binding site was identified based on the known binding position of the ligand. The GRID for docking was marked according to the ligand’s original binding site, using standard parameters in Sybyl-X. The receptor was kept rigid during docking, while the ligand was allowed to flex within the binding site. To validate the docking protocol, redocking was performed to ensure the ligand could dock successfully to its original position in the target’s binding site. The docked conformations were then evaluated based on their binding affinities and interactions with key amino acid residues.

The docking procedure involved several steps: first, the input chemical structure was optimized using Sybyl-X. The Tripos force field was then applied to minimize the molecules and assign Gasteiger–Hückel charges until convergence was reached (0.05 kcal/mol/Å). Next, the protein structure was processed in Sybyl-X by removing irrelevant ligands and solvent molecules, ensuring proper alignment for docking. Finally, PyMol software (http://www.pymol.org/pymol accessed on 15 June 2025) was used to visualize the docking results.

## 5. Conclusions

This study demonstrates the strong predictive performance and robustness of the LMIX3-SVR model, which integrates linear, polynomial, and RBF kernels for effective QSAR analysis of CatL inhibitors. The PSO algorithm optimized the model parameters, enabling rapid convergence and reducing computational time. Five new CatL inhibitors were designed based on key descriptors from the HM model, with compound 71d showing the most potent activity and the highest docking score (5.7972).

The designed inhibitors exhibited favorable binding affinities, particularly compound 71d, which suggests it as a promising high-affinity ligand for CatL. Toxicity predictions indicated medium risk. The compounds were also chosen for their synthetic feasibility, with predicted pharmacokinetic properties such as LogP, solubility, and drug likeness, suggesting good bioavailability and body distribution.

In conclusion, the LMIX3-SVR model proved effective in designing CatL inhibitors with promising pharmacological profiles, providing a strong foundation for further experimental validation and drug development to accelerate the discovery of safer, more effective anti-SARS-CoV-2 therapeutics.

## Figures and Tables

**Table 1:** The selected descriptors and their physical–chemical meanings and coefficient by HM.

Symbol	Physical–Chemical Meaning	Coefficient
RNR	Relative number of rings	−37.67
HDH2(QCP)	HA-dependent HDCA-2 (quantum-chemical PC)	0.20436
YS/YR	YZ shadow/YZ rectangle	−4.9021
MPPBO	Max PI-PI bond order	25.354
MEERCOB	Max e-e repulsion for a C-O bond	0.24247

**Table 2:** Correlation coefficient of the five descriptors by HM.

Descriptor	RNR	HDH2(QCP)	YS/YR	MPPBO	MEERCOB
RNR	1.00				
HDH2(QCP)	0.32	1.00			
YS/YR	−0.05	−0.33	1.00		
MPPBO	−0.09	0.25	−0.11	1.00	
MEERCOB	−0.58	−0.16	0.02	−0.16	1.00

**Table 3:** The selected descriptors and their physical-chemical meanings by XGBoost.

Symbol	Physical–Chemical Meaning
ABOCA	Avg bond order of a C atom
HDH2(QCP)	HA-dependent HDCA-2 (quantum-chemical PC)
HDH1(QCP)	HA-dependent HDSA-1 (quantum-chemical PC)
RNR	Relative number of rings
YS	YZ shadow
He	HOMO energy
MENACHB	Max e-n attraction for a C-H bond
MEERCOB	Max e-e repulsion for a C-O bond
YS/YR	YZ shadow/YZ rectangle
MPPBO	Max PI-PI bond order

**Table 4:** Correlation coefficient of the ten descriptors by XGBoost.

Descriptor	ABOCA	HDH2(QCP)	HDH1(QCP)	RNR	YS	He	MENACHB	MEERCOB	YS/YR	MPPBO
ABOCA	1.00									
HDH2(QCP)	0.36	1.00								
HDH1(QCP)	0.34	0.91	1.00							
RNR	0.77	0.32	0.16	1.00						
YS	0.67	0.14	0.01	0.80	1.00					
He	0.68	0.72	0.18	0.67	0.39	1.00				
MENACHB	−0.56	−0.74	−0.16	0.74	−0.61	−0.29	1.00			
MEERCOB	−0.67	−0.16	−0.62	−0.58	−0.63	−0.66	0.38	1.00		
YS/YR	0.58	−0.33	−0.65	−0.05	−0.09	0.66	−0.04	0.02	1.00	
MPPBO	0.42	0.25	0.76	−0.09	−0.11	−0.22	0.19	−0.16	−0.11	1.00

**Table 5:** Predicted IC_50_ by HM and Docking total score of new CatL inhibitors.

No.	CatL Inhibitors	Predicted IC_50_	Total Score
**71**	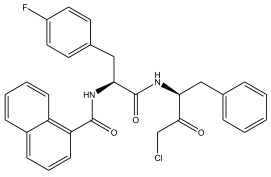	0.7006	4.6649
**71a**	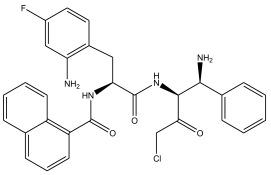	0.2305	4.8034
**71b**	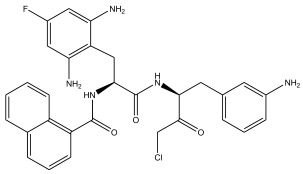	0.1992	5.3886
**71c**	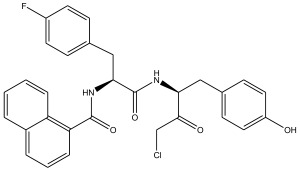	0.1541	5.5570
**71d**	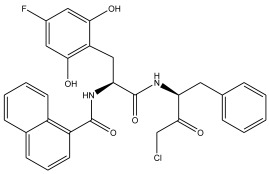	0.1556	5.7972
**71e**	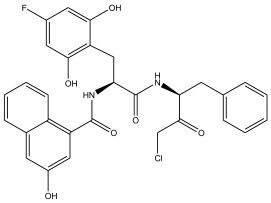	0.2589	5.7842

**Table 6:** Predicted IC_50_ by HM and properties by PEA of newly designed compounds.

No.	Pre.IC_50_	Toxicity	LogP	Solubility	Mol Weight	TPSA	Drug Likeness	Drug Score
**71**	0.7006	Medium	5.12	−6.96	516.0	75.27	4.55	0.09
**71a**	0.2305	Medium	2.97	−6.56	546.0	127.3	1.68	0.1
**71b**	0.1992	Medium	3.08	−7.18	561.0	153.3	1.47	0.09
**71c**	0.1541	Medium	4.77	−6.66	532.0	95.5	4.99	0.09
**71d**	0.1556	Medium	4.43	−6.36	548.0	115.7	4.08	0.1
**71e**	0.2589	Medium	4.08	−6.07	564.0	135.9	4.25	0.1

**Table 7:** y-Randomization results of different models.

Method	$\mathrm{Avg}\;R_{r}^{2}$	$\mathrm{Avg}\;R_{5-fold}^{2}$
HM	0.0274	−0.2760
RF	0.0493	0.0138
GEP	0.0962	0.0437
RBF-SVR	0.0873	0.0549
LMIX2-SVR	0.0787	0.0418
LMIX3-SVR	0.0526	0.0322

**Table 8:** Comparison of prediction and cross-validation results of different methods.

Statistical Parameters		HM	GEP	RF	RBF-SVR	LMIX2-SVR	LMIX3-SVR
$R^{2}$	Training set	0.8000	0.7637	0.9617	0.9431	0.9671	0.9676
	Test set	0.8159	0.7798	0.7781	0.8971	0.9410	0.9632
RMSE	Training set	0.0658	0.3394	0.1321	0.0063	0.0045	0.0834
	Test set	0.0764	0.2400	0.3089	0.0614	0.1199	0.0322
$R_{cv}^{2}$	$R_{5-fold}^{2}$	0.6139	0.7419	0.5163	0.8857	0.8914	0.9043
	$R_{LOO}^{2}$	0.7757	0.7826	0.7913	0.9236	0.9349	0.9525

**Table 9:** Comparison of statistical parameters of different methods.

Method	CCC	$Q_{F1}^{2}$	$Q_{F2}^{2}$	$Q_{F3}^{2}$
HM	0.8247	0.6269	0.6950	0.6344
GEP	0.8503	0.7064	0.6931	0.6988
RF	0.8890	0.7268	0.7931	0.7782
RBF-SVR	0.9290	0.9218	0.9427	0.9166
LMIX2-SVR	0.9725	0.9364	0.9428	0.9426
LMIX3-SVR	0.9839	0.9651	0.9793	0.9632

**Table 10:** Measured and predicted lg (IC_50_) of CatL inhibitors **1**–**42**. * The compounds of the test set.

	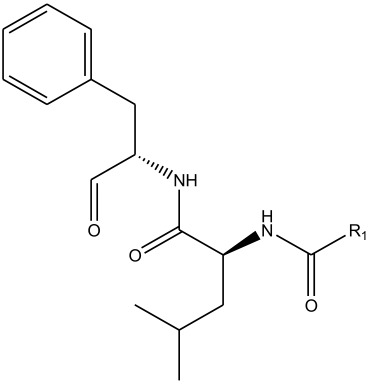							
Compound	R1	Measured lg (IC_50_)	HM	Predicted lg (IC_50_)				
				GEP	RF	RBF-SVR	LMIX2-SVR	LMIX3-SVR
**1**	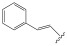	1.3539	1.8769	1.5765	1.5775	1.4044	1.3899	1.3280
**2** *	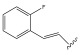	1.2172	1.8318	1.6091	1.3832	1.3613	1.3587	1.2271
**3**	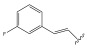	1.6648	1.8286	1.6228	1.7162	1.6508	1.6589	1.6613
**4**	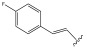	1.3023	1.8562	1.6006	1.5820	1.3874	1.3433	1.3021
**5**	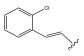	0.9809	1.1905	1.2188	1.1329	1.1151	1.1004	1.0808
**6** *	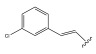	1.4328	1.6863	1.2439	1.4709	1.6367	1.5786	1.4557
**7**	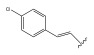	1.3711	1.7783	1.7174	1.5067	1.6322	1.4374	1.3608
**8**	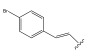	1.5841	1.7743	1.6398	1.6122	1.8608	1.7869	1.6008
**9**	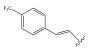	1.8794	1.8357	1.5132	1.7365	1.5005	1.6990	1.8533
**10** *	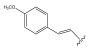	1.7434	1.914	1.5003	1.7169	1.6081	1.7304	1.7486
**11**	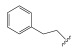	1.2594	1.4802	1.6057	1.4444	0.9779	1.1674	1.2876
**12**	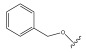	0.9948	1.3649	1.2156	1.0222	1.0224	1.0186	0.9930
**13**	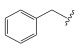	0.8932	1.1404	1.3617	0.9825	0.9476	0.9678	0.9301
**14**	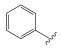	0.8785	1.1108	1.2717	0.9279	1.0320	0.9879	0.8645
**15**	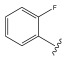	0.9849	1.0677	1.1991	0.9512	1.1483	1.0997	0.9721
**16**	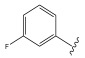	0.8751	0.8745	0.8636	0.9038	1.5166	1.1094	0.8703
**17** *	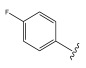	0.9934	0.9099	0.7741	0.9238	0.9228	0.9774	0.9979
**18**	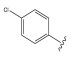	0.9763	0.9851	1.0689	0.9554	0.9832	1.0981	0.9734
**19**	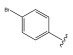	0.9818	1.0051	1.2399	0.9892	0.9535	1.2472	1.0132
**20**	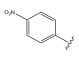	0.9133	1.0267	1.2828	0.9653	0.9239	1.7867	0.9094
**21**	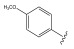	0.7825	1.0503	1.1018	0.9472	0.8651	0.8894	0.8019
**22** *	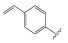	0.9736	1.0768	1.2927	0.8938	1.0321	0.9087	1.0471
**23**	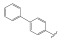	1.3209	0.5167	1.8394	1.0368	1.1483	1.2436	1.3208
**24**	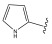	1.5767	1.4534	1.5829	1.0193	1.5166	1.5304	1.5665
**25**	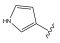	1.5715	1.4155	1.5366	1.5274	1.5912	1.6091	1.5738
**26**	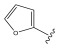	1.2395	1.2391	1.2836	1.5582	1.2088	1.2203	1.2394
**27**	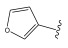	1.3842	1.0661	1.3334	1.2738	1.2890	1.3350	1.3657
**28**	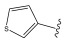	0.9112	0.8279	1.3169	1.2732	1.0678	0.9583	1.0011
**29**	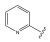	1.4219	1.284	1.3440	1.0047	1.3427	1.3342	1.4430
**30**	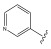	1.1599	1.1745	1.3821	1.3367	1.1093	1.1476	1.1656
**31**	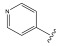	0.9274	1.1627	1.4359	1.1981	0.9882	0.9798	0.9336
**32**	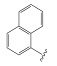	0.1004	0.427	0.4575	0.3949	0.4768	0.3354	0.1130
**33** *	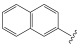	0.4518	0.3657	0.5971	0.5336	1.0167	0.8496	0.4496
**34**	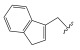	0.5024	0.7281	0.6151	0.5453	0.4377	0.4476	0.4867
**35**	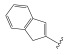	1.0056	0.8368	0.7854	0.8229	0.6235	0.8891	1.0758
**36**	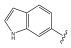	0.3118	0.5554	0.5922	0.6821	0.6859	0.4562	0.3003
**37** *	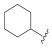	0.7825	1.2169	0.4556	0.8692	1.5106	0.9671	0.7796
**38**	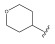	1.306	1.077	1.2009	1.1672	0.4914	0.9769	1.2968
**39**	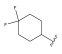	0.7987	1.3239	1.3294	1.0352	1.1345	0.8872	0.7886
**40**	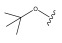	2.2163	1.7691	2.0254	2.0062	0.9565	1.8790	2.2109
**41**	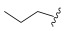	1.8613	1.6717	1.3951	1.6302	2.0201	2.3564	1.8513
**42** *	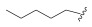	1.5396	1.6141	1.1529	1.5117	1.6651	1.5209	1.5387

**Table 11:** Measured and predicted lg (IC_50_) of CatL inhibitors **43**–**55**. * The compounds of the test set.

	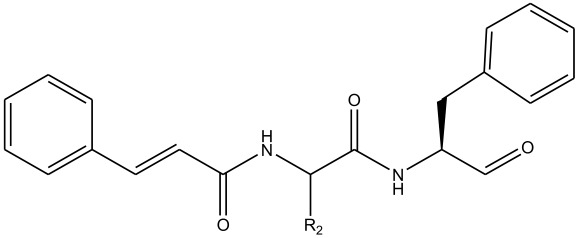							
Compound	R2	Measured lg (IC_50_)	HM	Predicted lg (IC_50_)				
				GEP	RF	RBF-SVR	LMIX2-SVR	LMIX3-SVR
**43** *	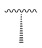	1.6245	1.327	1.4554	1.3328	0.9363	1.4327	1.6340
**44**	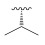	1.3432	1.5817	1.7364	1.1477	1.6907	1.5790	1.3342
**45**	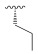	2.0952	1.9005	1.5272	1.9841	1.5856	1.8564	2.0962
**46**	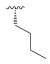	2.1962	1.9892	1.6842	2.0623	1.9925	1.9758	2.0987
**47**	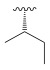	2.2301	1.7238	1.7872	1.8989	2.0298	2.1975	2.2185
**48**	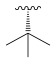	2.2611	1.8315	1.7062	2.0801	1.9854	1.8709	2.2408
**49**	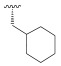	1.7239	1.0759	1.0428	1.1477	1.9640	1.8674	1.7098
**50** *	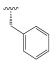	0.8228	0.8898	0.8122	0.8592	0.8565	0.9908	0.8325
**51**	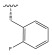	0.8338	0.9583	0.9013	0.8528	1.5941	0.7593	0.8567
**52** *	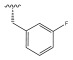	0.8169	0.9855	0.7423	0.8767	0.8438	0.7895	0.8067
**53** *	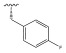	0.8248	0.7791	0.6758	0.8501	0.8563	0.8497	0.8032
**54**	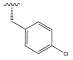	0.9499	0.8692	0.8135	0.9431	0.9502	0.9591	0.9366
**55**	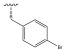	1.0626	1.0519	0.8721	1.1503	1.0353	1.0401	1.0598

**Table 12:** Measured and predicted lg (IC_50_) of CatL inhibitors **56**–**67**. * The compounds of the test set.

	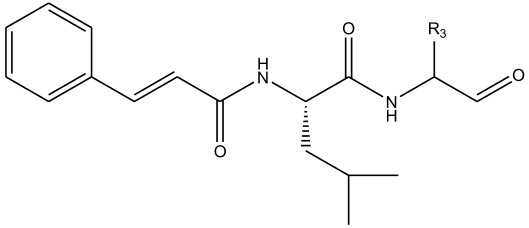							
Compound	R3	Measured lg (IC_50_)	HM	Predicted lg (IC_50_)				
				GEP	RF	RBF-SVR	LMIX2-SVR	LMIX3-SVR
**56**	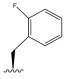	1.3685	1.9285	1.5641	1.6211	1.6011	1.4555	1.3587
**57**	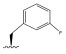	2.2106	1.8767	1.6683	2.0385	1.9226	2.0313	2.0986
**58** *	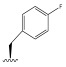	1.8696	1.4192	1.5753	1.6227	1.6769	1.7656	1.9008
**59**	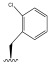	1.7634	1.8534	1.7065	1.7842	1.7952	1.7868	1.7437
**60**	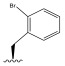	2.2555	1.7298	1.6850	1.6491	2.0879	2.1779	2.2037
**61**	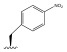	2.5374	2.0476	1.8594	1.9362	2.4385	2.6854	2.5768
**62**	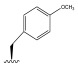	2.1164	1.8859	1.8139	2.2327	1.9688	2.0008	2.0983
**63**	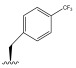	2.4036	2.0282	1.8618	1.6106	2.3779	2.3609	2.3906
**64**	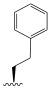	1.4146	1.2352	1.4064	1.1949	1.3479	1.6546	1.5603
**65**	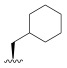	1.6791	1.3685	1.7389	1.4152	1.5973	1.8793	1.6372
**66** *	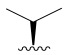	1.3696	1.5764	1.1939	1.4672	1.5425	1.4331	1.3870
**67**	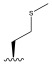	1.4678	1.4788	1.4711	1.1097	1.4695	1.4865	1.4574

**Table 13:** Measured and predicted lg (IC_50_) of CatL inhibitors **68**–**74**.

			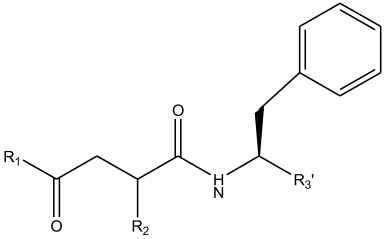							
Compound	R1	R2	R3’	Measured lg (IC_50_)	HM	Predicted lg (IC_50_)				
						GEP	RF	RBF-SVR	LMIX2-SVR	LMIX3-SVR
**68**	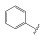	H	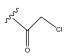	0.9943	0.8167	0.6589	0.4047	1.0670	1.2326	1.0942
**69**	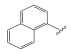	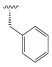	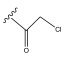	−0.3872	−0.3184	−0.0265	−0.2564	−0.3076	−0.3562	−0.3687
**70**	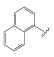	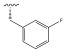	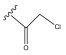	0.1271	−0.2058	0.2319	0.0712	0.0578	0.0945	0.1260
**71**	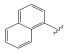	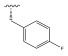	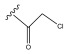	−0.5686	−0.1545	−0.0927	−0.1407	−0.3882	−0.6837	−0.4687
**72**	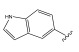	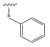	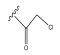	−0.0969	−0.2336	−0.1211	−0.0387	−0.1071	−0.1091	−0.1032
**73**	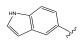	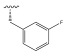	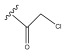	−0.1427	−0.0742	−0.2773	−0.1968	−0.1015	−0.1248	−0.1042
**74**	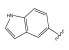	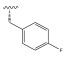	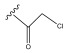	−0.2518	−0.2708	−0.1983	−0.1865	−0.1978	−0.1970	−0.2003

## Data Availability

The original contributions presented in this study are included in the article. Further inquiries may be addressed to the corresponding author.
